# Primary plasmacytoma of the cranial vault: a case report

**DOI:** 10.1186/1757-1626-2-9154

**Published:** 2009-12-07

**Authors:** Anrdeas Zigouris, Dimitrios Drosos, George A Alexiou, George Fotakopoulos, Evaggelos Mihos, Dimitrios Pahatouridis, Spyridon Tsiouris, Andreas D Fotopoulos, Spyridon Voulgaris

**Affiliations:** 1 Department of Neurosurgery, University Hospital of Ioannina, St Niarhou, Ioannina, 45500, Greece; 2 Department of Nuclear Medicine, University Hospital of Ioannina, St Niarhou, Ioannina, 45500, Greece

## Abstract

We report one case of a 78-year-old woman who referred to our hospital because of a progressive right hemiparesis. On clinical examination a painless large soft mass in the left parietal region was observed. CT and MRI revealed an extra-axial mass in the in the left fronto-temporo-parietal region. The lesion was totally excised despite the bleeding tendency. Histology disclosed the presence of a plasmacytoma. Postoperative, the patient developed an epidural hematoma that required immediate evacuation. On further investigation active tuberculosis was detected. On follow up examination 1 year later no tumor recurrence or evidence of multiple myeloma was detected.

## Introduction

Plasmacytes are responsible for the production of antibodies, consisting an important factor of the immune system. Plasmacytomas are referred to benign lesions that may progress to multiple myeloma, a fatal neoplasm [1]. Skull plasmacytomas are unusual tumors accounting for 4% of all plasma cell tumors [1-6]. We report on a rare case of a skull plasmacytoma in a patient with active tuberculosis and no evidence of multiple myeloma that was successfully treated by surgery alone.

## Case Report

### Presentation

A 78-year-old Caucasian woman from Greece was referred to our hospital because of a progressive weakness of the right upper and lower extremities and inability to walk. The clinical examination revealed a right hemiparesis, positive ipsilateral Babinski and Barré signs and a painless large soft mass in the left parietal region, which could be easily missed because of her long hair. The patient suffered from headache during the last 6 months, without alterations in mental status, as it was reported from her daughter.

### Diagnostic Tests

**Figure 1:**
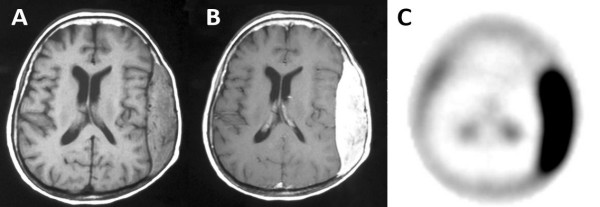
**A. Axial T1-weighted MR image revealing an isointense signal mass in the left temporoparietal region**. **B**. Marked enhancment after contrast administration. **C**. Reconstructed axial SPECT slice revealing significant ^99m^Tc-Tetrofosmin uptake in the left temporoparietal region.

A brain CT was performed and revealed an extradural mass that was homogenous enhance after intravenous contrast administration. There was bone erosion and the lesion had regions of calcifications. MRI that ensued showed a large (9.8 × 9 × 3 cm) extra-axial mass with isointense signal on T1-weighted images, hyperintense signal on T2- weighted images with intense and homogeneous contrast enhancement [Figure 1A, B]. The lesion infiltrated the adjacent bone without dura involvement, giving rise to the subcutaneous mass in the left fronto-temporo-parietal region. Perifocal edema in the left hemisphere and a midline shift of approximately 8 mm were also detected. For further investigation, brain SPECT with ^99m^Tc-Tetrofosmin was performed. There was increased radiotracer accumulation consisted with a lesion of high metabolic activity and vascularity [Figure 1C].

### Interventions

**Figure 2:**
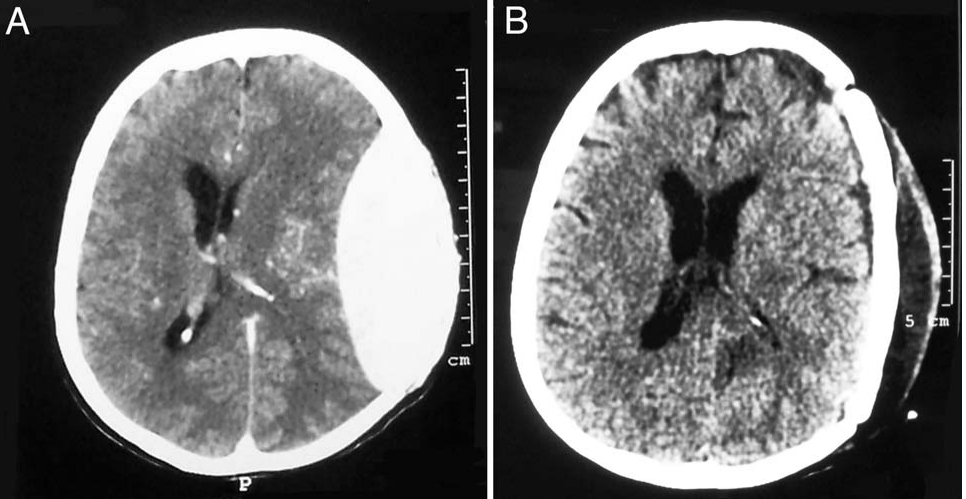
**A. Epidural hematoma six hours after plasmacytoma's excision**. **B**. Hematoma evacuation.

The patient was operated upon via a left fronto-temporo-parietal craniectomy. Intraoperative a soft purplish, easily separated from dural surface lesion was discovered that was not encapsulated and was originated from the diploe. The overlying skin was normal and the galea was not involved. There was a bleeding tendency, nevertheless the lesion was totally excised and a cranioplasty with bone cement was performed. Histology revealed a highly cellular tumor, composed of monoclonal plasma cells positive for immunoglobin lambda light chain. The diagnosis was plasmacytoma of the skull. Six hours later the patient developed an epidural hematoma that required an immediate evacuation [Figure 2]. The patient was admitted to the intensive care unit for four days. After readmission to neurosurgery department her neurological status was gradually improved with an accepted cosmetic appearance of the cranial vault. For further investigation to detect possible multiple myeloma a bone scan was performed that revealed no osteolytic lesions. On laboratory examination there was no evidence of systemic myelomatous changes, cancer or anemia. M component was not detected in serum protein electrophoresis, Bence-Jones protein and excretion of immunoglobin elements was not found in urine. The patient underwent bone marrow aspiration and bronchoscopy which were also negative. Saline cultures revealed active tuberculosis.

### Outcome

Postoperative the patient received treatment for tuberculosis (ethambutol, pyrazinamide and rifampicin for nine months) and adjuvant treatment for the plasmacytoma (thalidomide, melphalan and dexamethasone). One year after the initial diagnosis the patient was free of recurrence without having received additional radiotherapy.

## Discussion

Multiple myeloma has an incidence of 4 cases per 100.000/year and constitutes approximately 1% of all malignant neoplasms and 15% of all blood neoplasm's [3]. It is characterized by a decrease in the amount of beta-lymphocytes and the mean age of the patients is 60 years [1]. Plasmacytoma is a myelomatous mass that may be solitary, in combination with multiple myeloma or may progress to a generalized disease [7-9]. The diagnosis of solitary plasmacytoma can be made only when there is no evidence of multiple myeloma based on bone marrow aspiration, electrophoresis of serum and urine protein and no other lesion on complete skeletal survey [4,7]. Multiple myeloma predisposes to infections with atypical microbes including mycobacteria [10]. Our patient suffered from tuberculosis but there was no evidence of multiple myeloma.

Plasmacytoma of the skull is a rare finding [6]. It may involve the cranial vault, skull base and the orbit. Presenting symptoms and signs are not specific because plasmacytoma lacks neurological symptoms, except of cases of intraparenchymal dissemination or compression of brain and cranial nerves [9]. In that case symptomatology depends on the lesion's location [3]. Cosmetic skull deformities have been reported to be a usual cause for referring to a specialist [5]. In our case, except from the severe clinical deterioration, a problem of cosmetic appearance was also evident.

On radiological investigation CT and MRI may provide clues to narrow the differential [9]. We also performed a brain SPECT with ^99m^Tc-TF in order to functionally characterize the lesion. ^99m^Tc-TF is a lipophilic cationic diphosphine routinely used for myocardial perfusion imaging. Its whole uptake mechanism depends mainly on regional blood flow and cell membrane integrity [11]. In our case we observed an avid uptake of ^99m^Tc-TF that is explained by the high vascularity and metabolic activity of the tumor, but also by the lesion's absence of blood-brain barrier which allows leakage of the ^99m^Tc-TF from the cells. Nevertheless, further investigation is necessary to determine the usefulness of ^99m^Tc-TF brain SPECT in the diagnosis of this tumor type.

Total surgical resection followed by adjunctive radiation therapy has been advocated as an effective treatment in the majority of skull plasmacytomas [6]. Nevertheless, Arienta et al reported that if total resection has been achieved then radiotherapy should be reserved for case of tumor recurrence [2]. Furthermore, there are reports of complete cure after biopsy and radiotherapy, because plasma cell neoplasms are exquisitely radiosensitive [12]. In our case because of the complete tumoral excision, regular follow-up was preferred reserving radiotherapy for the future. Intraoperative, plasmacytoma may be a highly vascular tumor, therefore the neurosurgeon should be careful to perform a thorough hemostasis. A case of cardiac arrest from excessive blood loss has been reported [6]. In our case the tumor's bleeding tendency resulted in patient's reoperation due to an epidural hematoma. Additionally, based on our experience, we recommend craniectomy and cranioplasty, because there is a report of high recurrence rate from the remained cells of the tumor to the inner surface of the bone flap [3]. The reconstruction of the cranial vault improved also the cosmetic deformity.

To conclude, plasmacytoma of the cranial vault is a rare clinical entity that demands neurosurgeon's and hematologist's cooperation. Laboratory and imaging evaluation are of paramount importance to exclude systematic involvement. Careful surgical resection if total may be adequate for the disease control. Nevertheless, close follow-up with regular life-long examinations are important in order to avoid a generalized incurable disease.

## Abbreviations

CT: computed tomography; MRI: magnetic resonance imaging; ^99m^Tc-TF: ^99m^Tc-Tetrofosmin.

## Consent

Written informed consent was obtained from the patient for publication of this case report and accompanying images. A copy of the written consent is available for review by the Editor-in-Chief of this journal.

## Competing interests

The authors declare that they have no competing interests.

## Authors' contributions

SV and DP conceived of the study. AZ, GA and SV drafted the manuscript. AZ, DD, GF collected the data and performed the literature search. EM and DP are consultants in charge for the patient. ST and AF performed the SPECT study and analyzed the results. GA and EM participated in its design and coordination. GA, SV, AF and ST critical review the final version of the manuscript. All authors read and approved the final manuscript.
